# Detection of balance disorders using rotations around vertical axis and an artificial neural network

**DOI:** 10.1038/s41598-022-11425-z

**Published:** 2022-05-06

**Authors:** Marek Kamiński, Paweł Marciniak, Wojciech Tylman, Rafał Kotas, Magdalena Janc, Magdalena Józefowicz-Korczyńska, Anna Gawrońska, Ewa Zamysłowska-Szmytke

**Affiliations:** 1 Department of Microelectronics and Computer Science, Lodz University of Technology, 221 Wolczanska Str., 93-005 Lodz, Poland; 2 Audiology and Phoniatrics Clinic, Nofer Institute of Occupational Medicine, Lodz, Poland; 3 Balance Disorders Unit, Department of Otolaryngology, Medical University of Lodz, The Norbert Barlicki Memorial Teaching Hospital, Lodz, Poland

**Keywords:** Physical examination, Computer science

## Abstract

Vestibular impairments affect patients' movements and can result in difficulties with daily life activities. The main aim of this study is to answer the question whether a simple and short test such as rotation about a vertical axis can be an objective method of assessing balance dysfunction in patients with unilateral vestibular impairments. A 360˚ rotation test was performed using six MediPost devices. The analysis was performed in three ways: (1) the analytical approach based only on data from one sensor; (2) the analytical approach based on data from six sensors; (3) the artificial neural network (ANN) approach based on data from six sensors. For approaches 1 and 2 best results were obtained using maximum angular velocities (MAV) of rotation and rotation duration (RD), while approach 3 used 11 different features. The following sensitivities and specificities were achieved: for approach 1: MAV—80% and 60%, RD—69% and 74%; for approach 2: 61% and 85% and RD—74% and 56%; for approach 3: 88% and 84%. The ANN-based six-sensor approach revealed the best sensitivity and specificity among parameters studied, however one-sensor approach might be a simple screening test used e.g. for rehabilitation purposes.

## Introduction

Vestibular loss, even when compensated, affects patients’ movements, which can be observed as difficulties in the daily life activities. Vestibular impairment involves not only physical factors, but also emotional factors^1^, spatial cognitions and body representations^2^ and increases the risk of falling.

Vestibular information is mostly used to control orientation of the head and trunk in the space. This is particularly observed in patients with bilateral vestibular loss as the severe instability^3^. Lacour et al. reviewed the differences between static and dynamic compensation. The patients tend to improve after unilateral vestibular impairment, but the static balance recovery should be differentiated from the recovery of the dynamic one. The dynamic function observed during head or body movement^4^ can be recovered after sufficient time, however the improvement may be good but rarely complete. Mijovic et al.^5^ recognized that patients with a unilateral vestibular deficit took significantly longer to perform most daily life activities compared to controls because they adopted less efficient movement strategies. This has significant implications in vestibular rehabilitation and may be used in the assessment for legal purposes.

Posturography is the most useful method to assess the static balance, however this method does not reflect the ability to cope with dynamic conditions like walking or turning the body. There are several scales for functional assessment though they were not established exclusively for vestibular patients. Head movement or body turning is one of the tasks used in Timed-Up-and-Go test^6^, Sit-To-Stand test^7^, Dynamic Gait Index^8^, Berg Balance Scale^9^ and Tinetti Performance Oriented Mobility Assessment^10^.

The methods used for the assessment of balance dysfunctions require a special device (force plate) or consist of a clinical evaluation of several different physical activities. These tasks are often difficult to perform and may lead to falls. As none of these tests is without drawbacks, there is no gold standard in this field.

All these aspects led the authors to undertake a study to answer the question whether a simple and short test such as rotation about a vertical axis can be an objective method of assessing balance dysfunction. The issue of turning analysis has already been addressed in many studies albeit not in patients with vestibular deficit.

Mostly such studies referred to a specific disease entity (mainly Parkinson's disease)^11–18^. One of these studies^13^ examined head rotation and trunk rotation in patients with Parkinson's disease, whose straight-line gait is characteristically slow and short. The authors used the eight-camera Vicon 512 motion analysis system. Confirming clinical observations, patients with Parkinson's disease made slower and less efficient turns which required more steps. In another article^14^ subjects were asked to walk straight ahead for at least 2 m, make a left turn around the bar and continue walking in the new direction for at least 2 additional meters. The main abnormality detected was impaired intersegmental coordination, characterized by stronger than normal coupling of head and upper trunk rotation.

Turning the body is the essential challenge in the Timed Up & Go (TUG) test. In the study of post-stroke subjects with and without history of falls^15^ subjects who had suffered a stroke and experienced falls took significantly longer to perform a turn than age-matched control subjects. The task was monitored by 13 Vicon cameras at a sampling rate of 250 Hz.

There are papers concerning usage of the inertial measurement units (IMU) sensors, which includes 3-axis accelerometer and gyroscope, applied during the TUG test. In this test, the clinical measure is the total time in which the test is performed, while during the IMU test different parts (walking, turning, rising from a chair and sitting down) are recorded and may be evaluated separately. The turning time appeared to be more reliable in patients with Parkinson disease^19^. According to Zampieri et al.^20^ turning might be the most challenging part of the TUG test in Parkinson’s disease patients.

To the best of our knowledge there is limited literature data about using the rotation assessment in patients with vestibular deficit. Kim et al.^21^ published recently the first study on turning ability in group of patients with post-operational unilateral vestibular loss. In this study, acceleration and angular velocities along three perpendicular axes data were recorded from three IMUs placed on chest and each ankle during the TUG test. They analyzed turning and walking separately using the time of the task and the number of steps as variables and found the statistically significant slowing of the turn in patients with vestibular impairment as compared to healthy control. Kim et al. found that turning performance was significantly more impaired in UV patients than both the individuals with nonperipheral dizziness and healthy control. Moreover, in acute vestibular impairment it was just a turn, not a walk which improved after the rehabilitation. Gerhardy et al. in older population analyzed data from subphases of smartphone-based iTUG test and iClinical Test for Sensory Interaction on Balance (iCTSIB) based on IMU. They found that iTUG subphases of turning and walking were correlated with a lower vestibular performance in the iCTSIB.

The literature data presented above suggests that rotations taken into consideration alone have sufficient potential to diagnose the balance dysfunction.

The main purpose of this study is assessment of balance dysfunction in patients with unilateral vestibular impairments during the single rotation when using the single inertial sensor placed on the patient's body, or similar six sensors considered collectively. In the latter case, machine learning was employed, as it allows to combine data coming from various sources and seamlessly utilizes underlying relationships between data features, which may be crucial to the correct classification of the patient.

## Materials and methods

### Study group

The study group consisted of 102 persons, including 53 patients with verified balance dysfunction and 49 healthy persons. The characteristics of the groups and inclusion criteria are discussed below. The study protocol was approved by the Bioethics Committee. All participants signed informed consent form. All clinical examinations were conducted in line with the Declaration of Helsinki.

### Patients

The study included patients referred to the balance clinic because of vertigo and balance problems; at last 3 weeks after the acute vertigo onset. The detailed inclusion criteria included unilateral vestibular impairment confirmed by Videonystagmography caloric test (vestibular asymmetry > 20%) and symptoms confirmed by the symptoms questionnaires e.g. DHI and VSS. Every patient was able to perform the Romberg tests.

Age of patients varied between 29 and 84, with arithmetic mean of 58.2 (SD ± 14.8 years).

To compare the relationships between ipsi and contralateral direction of rotation and side of the lesion, the subgroup of 38 vestibular patients with deep vestibular asymmetry was extracted. The damage to the left side was observed in 21 patients and to the right side in 17 patients.

### Healthy persons

Age of healthy persons varied between 20 and 74, with arithmetic mean of 39.4 (SD ± 16.4 years). This group consisted of volunteers.

Absence of balance disorders was determined based on the following data.

- No history of dizziness, balance disorders, neurological disorders, disorders of circulatory system and musculoskeletal system, diabetes or migraines.

Any abnormalities results of physical examination, VNG test and dynamic posturography excluded the volunteer from inclusion into the healthy group.

### Methods

Measurement data was recorded using the prototype MediPost device. It is a portable, battery-powered, lightweight device controlled by the ESP32 system with a Wi-Fi radio module. It uses a 3-axis Inertial Measurement Unit (IMU) to determine its orientation in space. The IMU (STMicroelectronics LSM9DS1) contains a microelectromechanical system (MEMS) consisting of an accelerometer, a gyroscope and a magnetometer. The device is synchronized and controlled by a computer program. The application connects to a predefined Wi-Fi network via the defined port. Sampling frequency of 200 Hz is used on IMU device. Then a low-pass filter is implemented also on IMU, after which the signal can be represented with 20 samples per s. The samples (consisting of information taken from all three IMU sensors in three axes) are sent after the measurement is completed. The IMU Madgwick algorithm was chosen to determine the angular position of the device^22^. The algorithm is based on the use of quaternions to represent orientation in 3D space and treats the task of computing orientation as an optimisation problem, which it solves using a gradient descent approach. It was selected mainly because of its very low computational overhead, which is important in view of possible further work towards implementation of processing on a microcontroller-based platform. At the same time, it offers accuracy comparable to the Extended Kalman Filter^23^. Detailed description of the system can be found in^24,25^.

**Figure 1:**
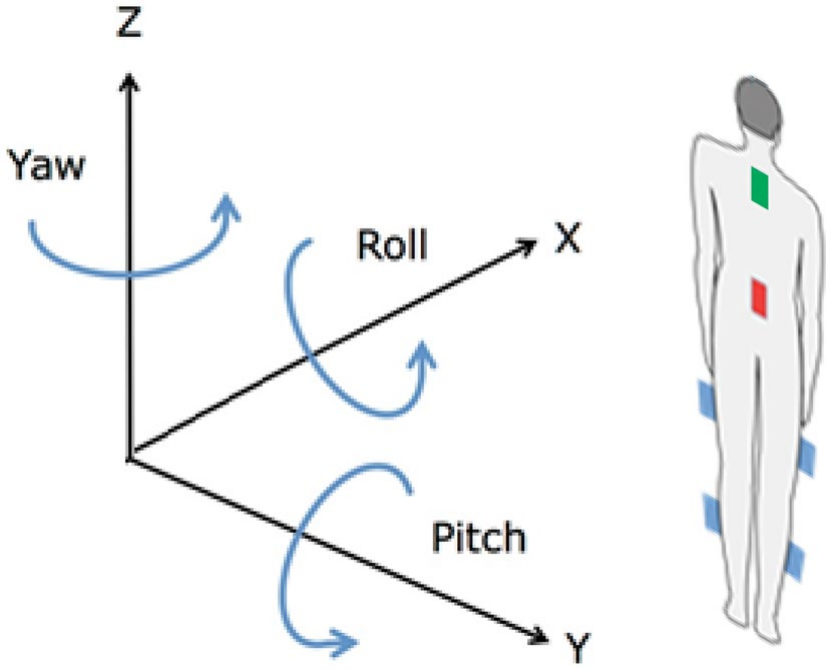
Placement of devices (coloured rectangles) on the body, various configurations: one-device—red or green, six-devices—red, green and blue.

Six MediPost devices are placed on the patient (Fig. 1). The analysis of the patient's rotation according to the single-sensor model focused on two sensors: mounted at the lumbar level (L5) and mounted on the nape of the neck. The choice was based on the fact that the easiest way to track rotation is to take into account the data from sensors located on the axis of rotation. The other four sensors are located on the patient's calves and thighs. These locations were selected as the patients’ legs perform relatively complex movements during the 360° rotation. Moreover, the patients were asked to keep their hands (another possible location for the sensors) along the body and not to perform unnecessary hand movements. Data from all six sensors were included in the analysis that employs the multi-sensor model.

The 360° counterclockwise rotation was taken into account as a single test. All patients in the study group and control were right-handed thus the left rotation was more natural in this group.

For patients with left vestibular impairment the rotation was ipsilateral while for right-side patients the rotation was contralateral to the site of the lesion. The ipsi and contralateral rotations were compared.

Before performing the tasks, all six sensors were calibrated in order to eliminate errors related to the drift of gyroscope sensors and to determine the position of the sensors in relation to the axis of rotation.

After hearing the beep signal, the patient made the rotation. All patients were able to perform full 360° rotation which could be included in the analyses.

### Data analysis

Two models were developed as part of the data analysis: a model based on data from a single sensor (L5) and a model based on data from six sensors.

### Single-sensor model

The result of Madgwick’s algorithm is the angular position of the sensor (pitch, roll and yaw angles) and the translation vector. Due to the fact that linear motions are not analysed, the translation vector is not used in further calculations. In order to track the progress of the rotation, authors mainly used the yaw angle. It is zeroed at the first time sample.

During observations of task execution, authors have noticed some patient behaviours that made analysis of the results more difficult.

- Slight turning of the body in the opposite direction to the correct one before the proper rotation.
- Rotation by an angle greater than 360°. The maximum overshoot of a full rotation was 40°.
- Correction of the position at the end of the rotation (when the subjects perceived their final position as inaccurate).

The start and the end moments of the rotation are calculated automatically. This contrasts the common practice of evaluating this type of exercise based only on medical observation (without a computer system). Stopwatch measurements of short exercise durations can be very inaccurate. Human error in capturing the moment of the start and the end of the rotation can significantly affect the results.

The conditions for detecting the start of a rotation are as follows:

- the value of yaw angle change is greater than 0.75° (within 0.05 s)
- a sample number is greater than 1

The conditions for detecting the end of a counterclockwise rotation are defined as follows:

- the value of yaw angle change is less than 0.75° (within 0.05 s)
- the value of yaw angle (measured from the start of the rotation) is greater than 300°
- the counterclockwise rotation has started

The limit values of angle change were chosen based on the measurement of several healthy, standing still (for 120 s) patients. For the calculations it was assumed that the task is performed correctly when the patient rotates between 300° and 360°. This was assumed because of errors in angle measurement and some inaccuracy in the patients' performance of the task. Requiring the rotation to be exactly 360° would lead to rejection of some exercises as incomplete. The rotation tracking scheme implemented in this way proved to be very effective.

A set of the following parameters was selected for analysis:

- maximum angular velocity—the value measured during exercise (expressed in degrees per second).
- average angular velocity—the value measured during rotation (expressed in degrees per second)
- latency—the duration from the beep sound to the start of rotation (expressed in seconds)
- rotation duration—the difference between the moment when rotation ends and the moment when rotation begins (value expressed in seconds)
- length of the trajectory defined by the projection of vertical axis of the sensor on the floor plane—the value expressed in millimetres and calculated after the elimination of rotation axis translations (Eq. 1)
  1 $$l\,=\, \text{H*}\sum_{i=2}^{N}\sqrt{{\left(\mathrm{sin}({pitch}_{i})-\mathrm{sin}({pitch}_{i-1})\right)}^{2}+{\left(\mathrm{sin}({roll}_{i})-\mathrm{sin}({roll}_{i-1})\right)}^{2}}$$

where H is the height of sensor mounting, N is the number of samples collected.

### Six-sensor model

The six-sensor model uses the same approach to compute the angular position of each sensor as the one-sensor design. This, in turn, allows to determine the angular position of body segments (after the calibration coefficients that describe the position of the sensor relative to the body segment are taken into account). The body segments are treated as rigid blocks connected with joints; such assumption together with the fact that at least one foot must touch the ground always allows to compute not only rotations, but also translations of the segments, thus re-creating body position in software.

The advantages of the six-sensor model, in comparison with the single-sensor one, are as follows:

- allows to compute movement parameters that appraise the body as a whole,
- is particularly useful in analysis of the movement of legs,
- may be used to perform more precise calibration procedures.

The last point is, however, also the source of a problem: the calibration procedure employed requires the patient to perform simple movements (a bow and a shallow squat) before performing the actual task. Unfortunately, in the analyzed study group not all of the subjects performed this procedure correctly. This reduced the group size for the six-sensor approach to 91 cases.

The following parameters have been proposed as a way to summarise body movements in the six-sensor approach. Unless otherwise specified, the parameter is computed based on the whole task, rotations are around the vertical axis of the world coordinates and the unit is degrees per second:

- latency, i.e., time from the buzzer indicating beginning of the task to the moment the patient started to rotate (in seconds),
- maximum angular velocity, taking into account all segments,
- maximum difference between momentary angular velocities of segments,
- maximum difference between momentary angular velocities of segments, relative to trunk segment,
- average angular velocity of trunk,
- maximum angular velocity of trunk,
- average sway velocity (i.e., angular velocity computed based on the angle between the vertical axis of the segment and the floor plane) of the trunk segment,
- maximum sway velocity of the trunk segment,
- rotation duration (in seconds),
- ratio of the average sway speed of the trunk segment to the average angular velocity of the trunk segment (unitless),
- ratio of the maximum sway velocity of the trunk segment to the maximum angular velocity of the trunk segment (unitless).

Analysis of the values obtained from the six-sensor model can be performed similarly to the one-sensor model, i.e., by selecting a single parameter and performing classification solely using such parameter. This approach has been presented in Table 3, which shows no benefit from the six-sensor approach over the one-sensor approach.

Another approach is to analyse them collectively. The authors argued that such approach may lead to better results, as it allows to combine parameters describing the task from different points of view. For this purpose, an Artificial Neural Network (ANN) was constructed using Keras library^26^. ANNs are bio-inspired computational models consisting of a number of simple unites—neurons—exchanging information with their neighbours. The neuron itself computes its output based on a weighted values of its inputs, further transformed by the so-called activation function. The employed network followed the Multi-Layered Perceptron concept, i.e., the neurons were organised into layers and the data flow was unidirectional and possible only between the adjacent layers. Various combinations of hyperparameters, such as network size, activation functions and learning algorithms were tested in order to find the best-performing networks. As overfitting (i.e., exhibiting good performance on the learning set with much poorer in case of samples that were not present in the set) is an important problem of ANNs, rigorous leave-one-out cross-validation was performed; the reported performance metrics are for the left-out samples.

### ROC curves

**Figure 2:**
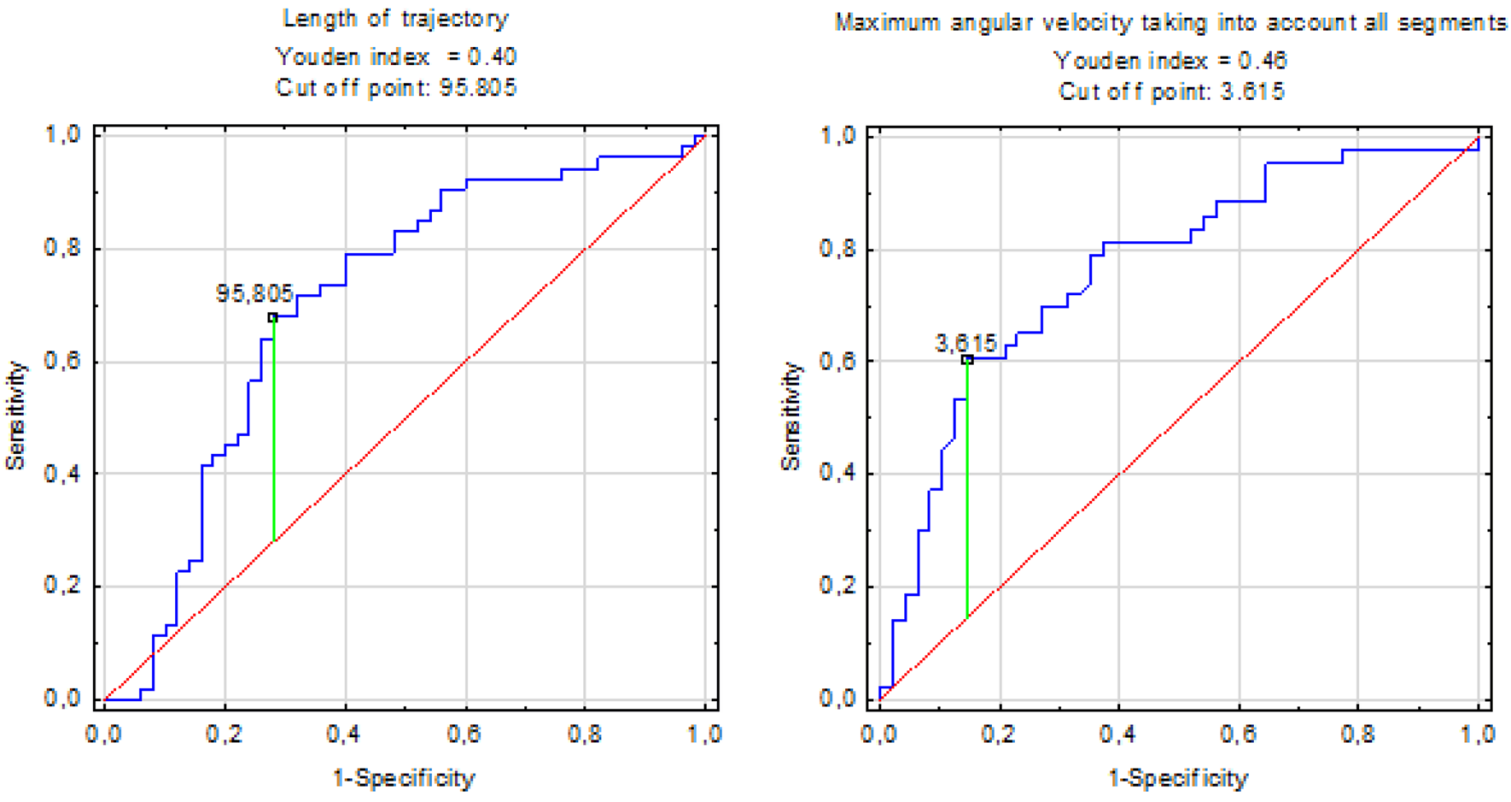
Example of obtained ROC curves.

When single parameters were analysed, ROC curves were used to determine the sensitivity and specificity of each approach. The area under the ROC curve (AUC) was calculated and statistical significance was set at 0.05. The cut-off point was calculated using the Youden index (Fig. 2). ROC curves were calculated for all listed parameters. Data were checked for normality with the Shapiro–Wilk test.

### Ethical standards

All procedures performed in studies involving human participants were in accordance with the ethical standards of the institutional and/or national research committee and with the 1964 Helsinki Declaration and its later amendments or comparable ethical standards. The study was approved by the Bioethics Committee of the Nofer Institute of Occupational Medicine, Lodz, Poland (No of protocol 17/2014).

## Results

### Single-sensor model

The first step of the analysis was to compare parameters obtained from the nape (Th_1_) sensor and from the L_5_ sensor. The values turn out to be very similar: maximum angular velocities differ on average by 5°/s (approximately 2.5%) and maximally by 20°/s; such differences are negligible. Rotation angle, which is particularly prone to the gyroscope drift error, differs on average by 20°—this value is similar to the observed inaccuracies introduced by the patients through imprecise execution of the task. It also does not affect the stop condition of the algorithm, as the task is considered to be performed correctly after the patient reaches 300th degree of the rotation.

For further computations data from the L_5_ sensor were selected—this sensor placement is more often used in imbalance analysis, it is also a place where sensor mounting is simpler.

The results for patients with a left-sided vestibular impairment were compared to the results for patients with a right-sided vestibular impairment. The rotation time and the maximum angular velocity were almost identical for both groups and did not show a statistically significant difference (for patients with left-sided defects t = 3.57 s, v_max_ = 176.1°/s; for patients with right-sided defects t = 3.57 s, v_max_ = 173.3°/s). This shows that for our study group the approach to choose only one direction of rotation for all patients was correct.

A comparison of the rotation parameters between the study group and the healthy patients is presented in Table 1.

**Table 1:** Comparison of healthy persons and unhealthy patients according to selected parameters.

Parameter	Healthy	patients	p-value
Maximum angular velocity	217.74 ± 55.52	173.64 ± 46.38	< 0.001
Rotation duration	2.79 ± 0.49 s	3.37 ± 0.97 s	< 0.001
Length of the trajectory	94.07 ± 46.46	111.39 ± 29.25	0.026

Table 2 shows the classification results based on the single-sensor model. The maximum angular velocity was the most sensitive parameter with the low specificity to differentiate between vestibular and healthy subjects.

**Table 2:** Single-sensor model results based on ROC analysis.

Parameter	AUC	p	Accuracy (%)	Sensitivity (%)	Specificity (%)
Maximum angular velocity	0.77	< 0.01	73	80	60
Average angular velocity	0.56	0.28	63	59	66
Rotation duration	0.75	< 0.01	72	69	74
Latency	0.59	0.12	61	29	91
Length of the trajectory	0.70	< 0.01	70	71	68

### Six-sensor analytical approach

Classification results for the six-sensor approach when a single parameter was selected for analysis are presented in Table 3; the analysis revealed no advantage over the one-sensor approach.

**Table 3:** Six-sensor model results based on ROC analysis.

Parameter	AUC	p	Accuracy (%)	Sensitivity (%)	Specificity (%)
Latency	0.59	0.13	58	81	37
Maximum angular velocity taking into account all segments	0.77	< 0.01	74	61	85
Average angular velocity taking into account all segments	0.66	0.01	65	72	58
Rotation duration	0.67	< 0.01	65	74	56

In the single parameter approach (e.g. when considering only the rotation time), the additional sensors did not provide much more information. The sensors on the neck and on the L4 behave almost identically in the measurements. Likewise, both sensors on the leg which did not coincided with the axis of rotation. Only in case of the leg which corresponded with the axis of rotation, differences could be observed. However, they were limited to the readings being slightly shifted in time, being ahead of the other sensors. The rationale for using six-sensor approach without in-depth analysis of minute variations in larger number of parameters comes down to increasing the accuracy of the measurement, by removing some of the errors (e.g., caused by extensive drift of one of the sensors).

### Six-sensor ANN-based approach

For the ANN-based six-sensor approach the network structure consisted of a single hidden layer with tanh activation function and an output layer with softmax activation function. This simple structure proved sufficient, neither more complex approaches, nor altering the activation function in the hidden layer resulted in better performance. Table 4 presents examples of results obtained for various combinations of hyperparameters. The best performing networks gave accuracy of over 80%, with one network reaching 86%.

**Table 4:** Six-sensor model classification results. Bold lines indicate best-performing networks.

Number of hidden layer neurons	Algorithm^A^	Number of epochs	Accuracy (%)	Sensitivity (%)	Specificity (%)
4	RMSprop 0.01, 0.7	100	78	83	73
4	**RMSprop 0.01, 0.7**	**200**	**84**	**83**	**84**
4	RMSprop 0.01, 0.7	400	82	83	82
5	RMSprop 0.01, 0.7	50	81	81	82
5	**RMSprop 0.01, 0.7**	**100**	**86**	**88**	**84**
5	RMSprop 0.01, 0.7	200	74	69	78
5	RMSprop 0.01, 0.7	1000	79	83	76
5	RMSprop 0.001, 0.7	100	80	81	80
5	**RMSprop 0.001, 0.7**	**200**	**84**	**81**	**86**
5	RMSprop 0.001, 0.7	400	80	81	80
6	RMSprop 0.01, 0.7	50	80	81	80
6	RMSprop 0.01, 0.7	100	76	76	76
6	**SGD 0.01, nesterov**	**200**	**84**	**83**	**84**
6	SGD 0.01, nesterov	400	80	81	80

^A^Algorithm name and parameters: learning rate and momentum coefficient/type.

## Discussion

The present study was designed to yield information if the single rotation test could be used for functional assessment of dynamic balance dysfunction in patients with unilateral vestibular weakness.

The studies were designed to check the impact of single-parameter and multi-parameter analysis based both on data from a single sensor and from multiple sensors placed on the patient's body. The Medipost system has been designed to easily collect data from several sensors at the same time. As the results show, data synchronously collected from a larger number of sensors allow for a more reliable reproduction of the movement mechanics, and at the same time allow to minimize the chance of measurement errors and inaccuracies in the patient's movements. However, particular parameters selected for analysis, calculated on the basis of six sensors, after eliminating the errors, are quite similar to the parameters from one sensor, so they do not carry much more diagnostic information. Only when a multi-parameter approach is used (in this case through fusing the parameters with the ANN) the collective impact of the minute differences is sufficient to noticeably improve accuracy.

In study group with not fully compensated vestibular impairment the results of rotation assessment were significantly worse than in healthy control. The study revealed that for balance assessment the ANN-based six-sensor approach was the most sensitive when compared to one-sensor and six-sensor approach with a single parameter selected for analysis (Table 5). Kim et al. published recently the first study on turning ability in group of patients with vestibular deficit^21^. They tested patients after schwannoma surgery with the complete unilateral vestibular (UV) loss. They found that during the turn UV patients were significantly slower than healthy controls which was the same observation as in our study. Moreover, turning takes more percent of time of total TUG than walking, which suggests that turning is more impaired than walking in UV patients. This observation is in the agreement with Dietrich et al. study^27^ which revealed that vestibular input may be selectively suppressed during fast locomotion. This proved the well-known observation that vestibular patients walk better faster than slowly. In TUG walking should be as fast as possible. Moreover, it was the turn which was the most improved after the rehabilitation in the Kim et al. study. The turn as a part of the TUG has also been investigated in patients with early Parkinson disease and has shown high accuracy^19^. Summarizing the single turn seems to be an effective task to reveal balance abnormalities in vestibular patients.

**Table 5:** Comparison of different methodologies for the analysis of rotation (the best results).

Parameter	Accuracy (%)	Sensitivity (%)	Specificity (%)
Maximum angular velocity, single sensor	73	80	60
Maximum angular velocity, taking into account all segments	74	61	85
Neural network taking into account all segments	86	88	84

The methodological aspects are very important when new method is introduced. Our study indicates that the unilateral character of the labyrinthine damage does not result in difference of measured parameters between turns ipsi- and contralateral to the impaired side. The similar results are presented in Kim et al. who found no differences in any kinematic variables for turns towards and away from the lesioned ear. Thus, one-side turn might have been chosen for testing.

Among the analyzed parameters, the most promising seem to be the duration of rotation and the maximum angular velocity. Although the rotation duration gives best performance and may be compared to iTUG analysis^21,28^, in our study the maximum angular velocity is the most reliable and resistant to errors in the task. Even interruptions of the task, improper termination or small errors in the gyro drift do not substantially deform its value. Their effectiveness, similar to that of static posturography^28^ suggests the possibility of using them for diagnostic purposes in patients with balance dysfunction. However, neither duration nor the maximum angular velocity result in high sensitivity and specificity while single-sensor model approach is chosen. The single-sensor test with the sensitivity of 80% and 60% specificity may be proposed as a simple screening test which confirms the balance abnormalities with lower specificity to vestibular background. According to Kim et al. rotation is the part of the TUG which markedly was improved during the rehabilitation.

Parameters based on averaged values (average speed and length of trajectory) appeared to be much worse predictors of imbalance (Table 2). This may be due to their lower resistance to any inaccuracies in the performance of the task, which appeared many times during the experiments, and also to gyroscope drift. The use of more sensors combined with the use of ANN to process the values obtained from them significantly improved the diagnostic capabilities of the entire approach up to sensitivity of 88 and 84% specificity. However, it should be noted, that this is a solution that requires the equipment which could be about six times more expensive (due to the multiplication of sensors) and significantly extends the diagnostic procedure—even though the patient performs the same task in the same way, the additional time necessary to conduct the test is longer (installing sensors, performing calibration, removing sensors).

## Conclusions

The conducted research is one of the first studies to confirm the clinical usefulness of rotations around vertical axis in assessment of the patients’ imbalance due to unilateral vestibular impairment. The test reached high sensitivity and specificity to reveal patients with not complete compensation after unilateral vestibular weakness. The ANN-based six-sensor approach revealed the best sensitivity and specificity among parameters studied however one-sensor approach might be a simple screening test used e.g. for rehabilitation purposes.

As part of future work, the authors plan to perform a similar analysis for another functional task—TUG, containing elements of rotation and also other types of movements. The authors would like to check how the multi-parameter analysis will affect the diagnostic properties of the TUG task.
